# Coordination Properties of the Fungal Metabolite Harzianic Acid Toward Toxic Heavy Metals

**DOI:** 10.3390/toxics9020019

**Published:** 2021-01-20

**Authors:** Gaetano De Tommaso, Maria Michela Salvatore, Rosario Nicoletti, Marina DellaGreca, Francesco Vinale, Alessia Staropoli, Francesco Salvatore, Matteo Lorito, Mauro Iuliano, Anna Andolfi

**Affiliations:** 1 Department of Chemical Sciences, University of Naples Federico II, 80126 Naples, Italy; gaetano.detommaso@unina.it (G.D.T.); mariamichela.salvatore@unina.it (M.M.S.); dellagre@unina.it (M.D.); frsalvat@unina.it (F.S.); 2 Council for Agricultural Research and Economics, Research Centre for Olive, Fruit and Citrus Crops, 81100 Caserta, Italy; rosario.nicoletti@crea.gov.it; 3 Department of Agricultural Sciences, University of Naples Federico II, 80055 Portici, Italy; alessia.staropoli@unina.it (A.S.); matteo.lorito@unina.it (M.L.); 4 Department of Veterinary Medicine and Animal Productions, University of Naples Federico II, 80137 Naples, Italy; frvinale@unina.it; 5 Institute for Sustainable Plant Protection, National Research Council, 80055 Portici, Italy; 6 BAT Center—Interuniversity Center for Studies on Bioinspired Agro-Environmental Technology, University of Naples Federico II, 80055 Portici, Italy

**Keywords:** Trichoderma, secondary metabolites, abiotic stress, metal-chelating properties

## Abstract

Some Trichoderma strains are known for their capacity to produce harzianic acid, a metabolite belonging to the tetramic acid derivatives. Harzianic acid has interesting biological properties, such as antimicrobial activities against phytopathogenic fungi and promotion of plant growth. It also possesses remarkable chemical properties, including the chelating properties toward essential transition metals, which might be related to the biological activities. Increasing knowledge on chelating properties might be relevant for understanding the various beneficial effects of harzianic acid in the interaction between the producer fungi and plants. In this work, the coordination capacity of harzianic acid was studied to evaluate the formation and stability of complexes formed with toxic heavy metals (i.e., Cd^2+^, Co^2+^, Ni^2+^, and Pb^2+^), which might have a crucial role in the tolerance of plants growing in metal-contaminated soils and in abiotic stress.

## 1. Introduction

Heavy metals are present in different concentrations in many rocks and soils, but the major sources of these elements are anthropogenic activities, including industrial emissions and agricultural treatments [1]. Some heavy metals are essential elements for physiological functions, e.g., iron (Fe), copper (Cu), zinc (Zn), and manganese (Mn), while many of them are commonly considered as toxic elements to plants and humans, e.g., lead (Pb), cobalt (Co), cadmium (Cd), nickel (Ni), chromium (Cr), mercury (Hg) [2]. There is ample literature on the acute and/or chronic toxic effects of these heavy metals for plants and animals [3,4,5]. For instance, in humans, nickel can have an impact on the immune, respiratory, nervous, and reproductive systems [6]; cobalt is mainly responsible for neurological, cardiovascular, and endocrine deficits [7]. Cadmium intoxication can lead to kidney, bone, and pulmonary damages [8]. Finally, lead exposure can cause an increase of oxidative stress, producing various deleterious effects on the hematopoietic, renal, reproductive, and central nervous systems [9].

Rapid industrialization and urbanization have caused environmental contamination and pollution by toxic heavy metals, and their concentrations above threshold levels (which are defined by national guidelines) can have negative effects on the ecological health and fertility of soils due to their bioaccumulation [10]. Bioavailability of heavy metals is mainly affected by their total content in soil and by the presence of microbial and plant species [11]. In this respect, fungi can contribute to increase the tolerance of plants growing in metal-contaminated soils, and the production of metal-chelating compounds has been demonstrated in some fungi, such as Pleurotus ostreatus [12], Beauveria caledonica [13], and Aspergillus niger [14]. In fact, organic acids may affect potentially toxic metals desorption, solubility, and mobility through the formation of complexes [15,16]. The most frequent fungal organic acids involved in the complexation of heavy metals in soils are oxalic, succinic, tartaric, and citric acids, which have the valuable capacity to remove heavy metals without destroying the soil matrix [17,18]. Moreover, several fungal genera (e.g., Saccharomyces, Aspergillus, and Penicillium [19]) produce a wide range of siderophores, which have strong affinity for ferric ion, but despite their preference for iron, they can chelate numerous metals (e.g., aluminum, cadmium, cobalt, silver, copper) with diverse affinity, having an impact on the transport and tolerance of these metal ions [20,21]. Hence, the heavy metals chelation by organic acids may represent an important detoxification mechanism, both by the fungi and plants [22], but it is dependent upon metals, ligands, and environmental conditions [23].

Beneficial microbes belonging to the genus Trichoderma are model organisms to study plant-microbe interactions, and some selected strains are present as active ingredients in bioformulations for agriculture [24,25]. Methods for remediating polluted soils or water, and for removing toxic substances using Trichoderma spp. in plant-microbes systems have been proposed [26]. These microbes are known to control plant pathogens and improve plant growth, also due to the production of a remarkable range of secondary metabolites showing different biological activities [25,26,27,28,29,30,31,32,33].

One of these effector metabolites is harzianic acid, a natural compound with notable biological activities and interesting chemical properties, including affinity to ferric iron, antimicrobial activity (e.g., Staphylococcus pseudintermedius, Pythium irregulare, Sclerotinia sclerotiorum, and Rhizoctonia solani) and promotion of plant growth (e.g., tomato) [34,35,36,37]. It has been recently obtained by total synthesis [38], and classified in the subgroup of the dienoyl tetramic acids [39].

In this work, the capacity of harzianic acid, obtained from cultures of a marine-derived strain of Trichoderma pleuroticola [40], to complex some dipositive toxic heavy metals (i.e., Cd^2+^, Co^2+^, Ni^2+^ and Pb^2+^), was studied. Several techniques have been used (i.e., mass spectrometry (MS), circular dichroism (CD) spectrometry, nuclear magnetic resonance (NMR), Fourier transform-infrared spectroscopy (FT-IR)) either to demonstrate qualitatively the capacity of harzianic acid to complex the targeted cations or to obtain clues on the coordination mode, although the stoichiometry and formation constants of complex species, in a CH_3_OH/0.1 M NaClO_4_ (50:50 *w*/*w*) mixed solvent at 25 °C, were ultimately obtained from UV-VIS spectrophotometric data collected in a wide pH and spectral range.

## 2. Materials and Methods

### 2.1. Reagents and Their Analysis

Analytical grade products Pb(ClO_4_)_2_, Cd(ClO_4_)_2_, CoCl_2_, and NiCl_2_ Merck (Darmstadt, Germany) were used to prepare stock aqueous solutions of metals whose concentrations were determined by complexometric titration with a standard solution of EDTA. Solutions of perchloric acid were prepared from analytical grade products and standardized potentiometrically (glass electrode) against tris(hydroxymethyl)amino methane (Sigma-Aldrich, Saint Louis, MO, USA). The results agreed to within 0.1% or better. Carbonate-free NaOH and NaClO_4_ solutions were prepared as previously described [40].

Harzianic acid was isolated as product of Trichoderma pleuroticola L1 recovered from a specimen of the mollusc Melarhaphe neritoides (Gastropoda, Littorinidae) collected along the coastline of the isle of Procida, Bay of Naples, Italy. The isolation and identification of this compound were previously explained in details [40]. Briefly, culture filtrate of T. pleuroticola was exhaustively extracted in acid conditions with ethyl acetate. The residue obtained was dissolved in chloroform and extracted with a saturated solution of NaHCO_3._ The aqueous phase was acidified and extracted with ethyl acetate to obtain a residue identified as harzianic acid, comparing the NMR data with previous reports [38].

### 2.2. Preparation of Test Solutions, Potentiometric, UV-VIS, CD, NMR, and IR Measurements

Test Solutions (TS), for CD and UV-VIS spectrometry, were prepared in an air-bath thermostat kept at (25.00 ± 0.05) °C by the procedure described in Section 3.

Potential of cell −RE/TS/GE+, which serves to evaluate the pH (=−log[H^+^]) of Test Solutions for UV-VIS and CD spectrometric measurements, was measured at 25.0 °C with a precision of ±0.01 mV using a Keithley 642 type Digital Electrometer (Tektronix Inc., Beaverton, OR, USA). The pH indicator glass electrodes (GE) were Metrohm (Herisau, Switzerland) of 60102-100 type and an Ag/AgCl(s)/0.1 M NaCl/(0.1 M NaClO_4_/CH_3_OH, 50/50 w/w) electrode was utilized as reference (RE).

UV-VIS spectra were recorded by Cary model 5000 Spectrophotometer by Varian C. (Palo Alto, CA, USA), from 200 to 600 nm (optical path 0.2 cm) at 25.0 °C, under a constant flow of nitrogen.

The far UV-CD spectra were recorded with a JASCO CD spectrometer model J-715 (Tokyo, Japan), from 200 to 600 nm (optical path 0.2 cm) at 25.0 ° C, under a constant flow of nitrogen.

NMR spectra were recorded at 400 MHz in CD_3_OD/D_2_O 1:1 (*w/w*) on a Bruker spectrometer (Ascend^TM^400) (Bremen, Germany). The solvent was used as internal standard.

FT-IR spectra were recorded in modality attenuated total reflectance (ATR) with model Nicolet 5700 by Thermo Electric Corporation (Waltham, MA, USA). The measuring cell consisted of a mono crystal of zinc selenide. The blank was recorded using air as reference. The solid compounds, metal-harzianic acid, were prepared by mixing and evaporating equimolar solutions of the individual components.

### 2.3. HPLC-MS Analyses

HPLC-MS analyses were done with the methods described by [40] on an Agilent high performance liquid chromatograph (HPLC) 1260 Infinity Series (Agilent Technologies, Santa Clara, CA, USA) coupled to a quadrupole-time of flight (Q-TOF) mass spectrometer model G6540B (Agilent Technologies) with a Dual ESI source (Agilent Technologies). Briefly, 500 μL of a 2 mM solution (in water) of each metal (Cd(ClO_4_)_2_, CoCl_2_, NiCl_2_ and Pb(ClO_4_)_2_) were mixed with 500 μL of harzianic acid (solubilized in methanol, 1 mg·mL^−1^) and directly infused into the LC-MS system (7 μL injection volumes, eluted with 0.1% formic acid in acetonitrile at a flow rate of 0.3 mL·min^−1^ to the mass spectrometer). The system was operated in positive ion mode and a standard solution of purine and hexakis(1H,1H,3H-tetrafluoropentoxy)-phosphazene was infused to obtain the real-time lock mass correction. All parameters and acquisitions were set using the Agilent MassHunter Data Acquisition Software, rev. B.05.01 (Agilent Technologies).

## 3. Results and Discussion

**Figure 1:**
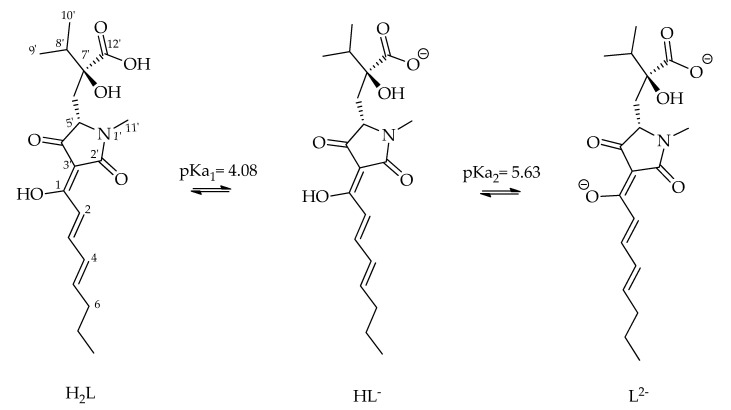
Structures of harzianic acid (H_2_L) and its acid dissociation products (HL^−^ and L^2−^). Reported dissociation constants of harzianic acid have been separately determined at 25 °C in the mixed solvent CH_3_OH/0.1 M NaClO_4_ (50:50 *w/w*), which is the solvent employed in this study.

Since harzianic acid is a diprotic acid, in the following text we use the abbreviation H_2_L, which represents the fully protonated species. Dissociation of protons by H_2_L produces in sequence HL^−^ and L^2−^, according to the dissociation constants exposed in Figure 1 [40]. In addition, when appropriate, the symbol M^2+^ will be used to generically represent the dipositive metal cations investigated in this study, i.e., Cd^2+^, Co^2+^, Ni^2+^, and Pb^2+^.

A rather unconventional approach has been used in this study to investigate the capacity of harzianic acid to complex bivalent heavy metal cations, M^2+^. In fact, in order to obtain clues on the formation and stoichiometry of complexes eventually present in solutions of the metal cations and harzianic acid, we first collected LC-ESI-HRMS of solutions of harzianic acid in the presence, respectively, of Cd^2+^, Co^2+^, Ni^2+^, and Pb^2+^.

To this aim, solutions of each M^2+^ cation and harzianic acid were prepared by mixing 500 μL of 2 mM solution of each metal cation in water (prepared by dissolving Cd(ClO_4_)_2_, CoCl_2_, NiCl_2_, and Pb(ClO_4_)_2_, respectively) with 500 μL of 1 mg·mL^−1^ harzianic acid in methanol. Finally, 7 μL of each solution were directly infused into the LC-MS system operated in the positive ion mode.

The most abundant ions in MS of each solution (Figures S1–S4) are reported in Table 1. From Table 1, it can be seen that, in the collected MS, a well-developed MS peak can be detected whose accurate mass corresponds to the composition [H_3_L_2_ + Metal]^+^. Furthermore, in the case of Pb^2+^, a complex ion of composition [HL + Pb]^+^ was also detected.

Based on our experience, MS data in Table 1 represent a conspicuous indication that complex species of the same or different stoichiometry may exist in solution.

In order to provide evidence for complex formation taking place in solution between targeted M^2+^ cations and harzianic acid, and to eventually ascertain their stoichiometry and evaluate their formation constants, circular dichroism (CD) and UV-VIS spectra of solutions of the M^2+^ cations and harzianic acid were acquired in a wide pH and spectral range.

Because of the low solubility of harzianic acid in water, CH_3_OH/0.1 M NaClO_4_ (50:50 *w/w*) was employed as solvent. The constant concentration of NaClO_4_ in the solvent is high enough, with respect to the reagent concentrations employed in this study, to provide a constant ionic strength in all of the investigated solutions. So much so that activity coefficients of reagents and products of complex formation reactions can be considered constant, and equilibrium constants can be expressed using concentrations in place of activities. In this context, the standard symbol pH merely indicates the molar concentration of solvated protons into the tested solutions (i.e., briefly, pH = −log[H^+^]).

**Scheme 1:**
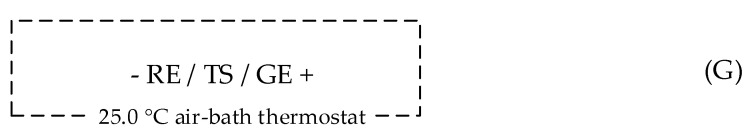
Sketch of the potentiometric apparatus employed to prepare test solutions, TS, of accurately known pH and analytical composition. RE represents the double junction Ag/AgCl(s)/0.1 M NaCl/(0.1 M NaClO_4_-CH_3_OH, 50/50 *w/w*) reference electrode and GE a pH indicator glass electrode.

Preparation of test solutions (TS), of accurately known pH and analytical composition, of each metal cation and harzianic acid, was performed at 25.0 °C, in a potentiometric multi-neck titration cell equipped with an Ag/AgCl(s)/0.1 M NaCl/(0.1 M NaClO_4_-CH_3_OH, 50/50 *w/w*) double junction reference electrode (RE) and a pH indicator glass electrode (GE) (Scheme 1).

Because of the large concentration of NaClO_4_ in the salt bridge of the double junction reference electrode and in the test solution, TS, the potential of cell (G) can be expressed, at 25 °C, by the simple Equation (1), in which $E_{G}^{0}$(volt) is a constant that was determined before each experiment by ad hoc potentiometric measurements.

(1) $$E_{G}\;={\;E}_{G}^{0}\;+\;0.05916\cdot \log\left[H^{+}\right]$$

In fact, in order to assess the value of $E_{G}^{0}$, each experiment started with a measured volume of pure solvent (i.e., CH_3_OH/0.1 M NaClO_4_ (50:50 *w/w*)) in the titration vessel. The potential, E_G_ (volt) of cell (G) was measured after stepwise addition of measured volumes of a standard solution of HClO_4_ in the same solvent, and the collected data were employed to evaluate ${\;E}_{G}^{0}$. After this initial glass electrode calibration procedure, pH (= −log[H^+^]) of any test solution in the potentiometric vessel can be evaluated from the measured potential, E_G_ (volt) of cell (G), at 25.0 °C, using the simple Equation (2):

(2) $$\mathrm{pH}=-\log\left[H^{+}\right]=\;\frac{E_{G}^{0}-E_{G}}{0.05916}$$

Subsequently, to the acidic solution in the titration vessel of cell (G), measured volumes of stock solutions of the metal cation and of harzianic acid were added so that the solution in the potentiometric vessel assumed the general composition specified by the analytical array (3).

(3) $$\left\{C_{H_{2}L}^{0}{\;M\;H}_{2}L+{\;C}_{M}^{0}\;M\;M{\left({\mathrm{ClO}}_{4}\right)}_{2}+{\;C}_{H}^{0}{\;M\;\mathrm{HClO}}_{4}\right\}$$

Then, the resulting solution (whose pH was accurately known), was sampled by withdrawing an accurately measured volume, which was transferred to the spectrometer cuvette (0.2 cm optical path) and processed at 25 °C.

After this, the pH of the solution in the titration vessel of cell (G) was systematically modified by successive additions of small volumes of a standard solution of NaOH, in the solvent CH_3_OH/0.1 M NaClO_4_, so that it assumed the general analytical composition specified by the analytical array (4), which could be accurately calculated from the preparation data.

(4) $$\left\{C_{H_{2}L}{\;M\;H}_{2}L+{\;C}_{M}\;M\;M{\left({\mathrm{ClO}}_{4}\right)}_{2}+{\;C}_{H}{\;M\;\mathrm{HClO}}_{4}+{\;C}_{\mathrm{NaOH}}\;M\;\mathrm{NaOH}\;\right\}$$

After each addition of NaOH, samples of the solution, whose pH could be obtained from Equation (2), were withdrawn from the titration vessel, transferred to the spectrometer cuvette and processed at 25 °C.

Obviously, all of the successive solutions prepared by this procedure share the same ligand to metal ratio, which keeps equal to the initial ratio $C_{H_{2}L}^{0}/C_{M}^{0}$, while the metal cation and harzianic acid concentrations, $C_{H_{2}L}$ and C_M_, respectively, in the withdrawn samples, decrease as their pH increases.

**Figure 2:**
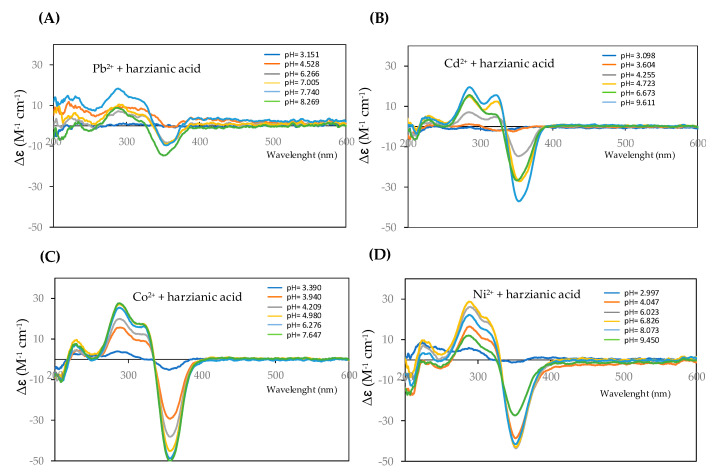
Far–UV circular dichroism (CD) spectra (optical path 0.2 cm) in CH_3_OH/0.1 M NaClO_4_ (50:50 *w/w*) at different pH values: (**A**)$\;C_{\mathrm{Pb}}^{0}$ = 5.4 × 10^−5^ M Pb(ClO_4_)_2_ and $C_{H_{2}L}^{0}$ = 1.1 × 10^−4^ M; (**B**)${\;C}_{\mathrm{Cd}}^{0}$ = 1.4 × 10^−4^ M Cd(ClO_4_)_2_ and $C_{H_{2}L}^{0}$ = 2.6 × 10^−4^ M; (**C**) $C_{\mathrm{Co}}^{0}$ = 6.9 × 10^−5^ M CoCl_2_ and $C_{H_{2}L}^{0}$= 1.4 × 10^−4^ M; (**D**) $C_{\mathrm{Ni}}^{0}$ = 1.1·10^−4^ M NiCl_2_ and $C_{H_{2}L}^{0}$ = 2.1 × 10^−4^ M. H_2_L = harzianic acid.

The acquired CD spectra are exposed in Figure 2.

Inspection of CD spectra shows the presence of two peaks whose intensity increases by increasing pH. One positive peak at 285 nm (Cotton effect) is attributed to transitions involving the diene system orbitals, and the second negative peak at 350 nm is associated to transitions that involve the non-bonding electrons of oxygen in C4’ position of harzianic acid [40].

Qualitatively, similar effects are also observed in solutions of only harzianic acid, but the dependence of CD spectra on the specific metal cation undoubtedly points toward specific interactions between M^2+^ cations and the ligand.

Next, UV-VIS spectrophotometric data, used for the evaluation of the stoichiometry of complexes and of their formation constants, were collected.

For each metal, two spectrophotometric datasets were acquired, by fine-tuning the initial addition of the harzianic acid and metal cation stock solutions, to the acidified solvent in cell (G), so that $C_{H_{2}L}^{0}$ = 2.0 × 10^−4^ M and $C_{M}^{0}$ = 2.0 × 10^−4^ M (i.e., 1:1 ligand to metal ratio) or $C_{H_{2}L}^{0}$ = 2.0 × 10^−4^ M and $C_{M}^{0}$ = 4.0 × 10^−4^ (i.e., 1:2 ligand to metal ratio).

**Figure 3:**
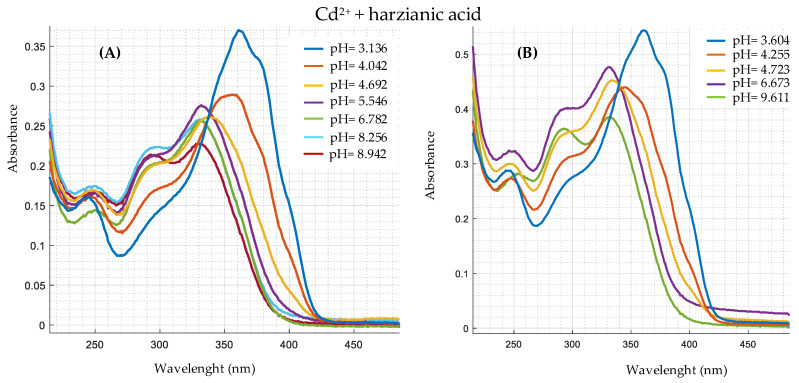
UV-VIS spectra in CH_3_OH/0.1 M NaClO_4_ (50:50 *w/w*) at different pH of: (**A**) $C_{H_{2}L}^{0}$ = 2.0 × 10^−4^ M and $C_{\mathrm{Cd}}^{0}$ = 2.0 × 10^−4^ Cd(ClO_4_)_2_ (ligand to metal ratio 1:1); (**B**) $C_{H_{2}L}^{0}$ = 2.0 × 10^−4^ M and $C_{\mathrm{Cd}}^{0}$ = 4.0 × 10^−4^ Cd(ClO_4_)_2_ (ligand to metal ratio 1:2).

**Figure 4:**
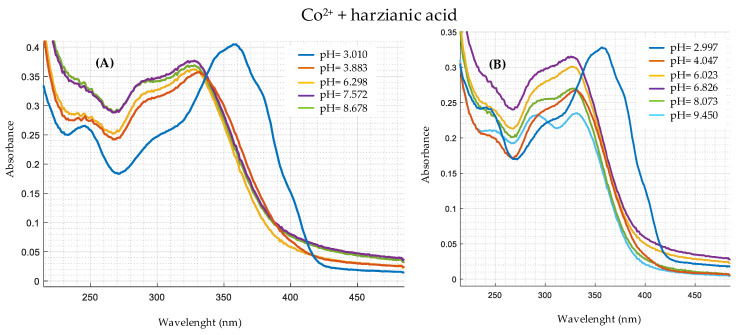
UV-VIS spectra in CH_3_OH/0.1 M NaClO_4_ (50:50 *w/w*) at different pH of: (**A**) $C_{H_{2}L}^{0}$ = 2.0 × 10^−4^ M and $C_{\mathrm{Co}}^{0}$ = 2.0 × 10^−4^ CoCl_2_ (ligand to metal ratio 1:1); (**B**) $C_{H_{2}L}^{0}$ = 2.0 × 10^−4^ M and $C_{\mathrm{Co}}^{0}$ = 4.0 × 10^−4^ CoCl_2_ (ligand to metal ratio 1:2).

**Figure 5:**
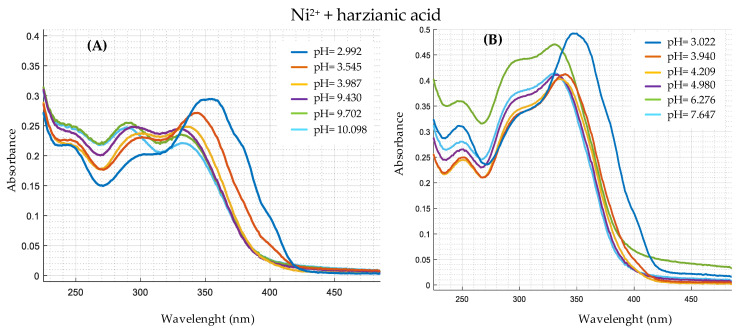
UV-VIS spectra in CH_3_OH/0.1 M NaClO_4_ (50:50 *w/w*) at different pH of: (**A**) $C_{H_{2}L}^{0}$ = 2.0 × 10^−4^ M and $C_{\mathrm{Ni}}^{0}$ = 2.0 × 10^−4^ NiCl_2_ (ligand to metal ratio 1:1); (**B**) $C_{H_{2}L}^{0}$ = 2.0 × 10^−4^ M and $C_{\mathrm{Ni}}^{0}$ = 4.0 × 10^−4^ NiCl_2_ (ligand to metal ratio 1:2).

**Figure 6:**
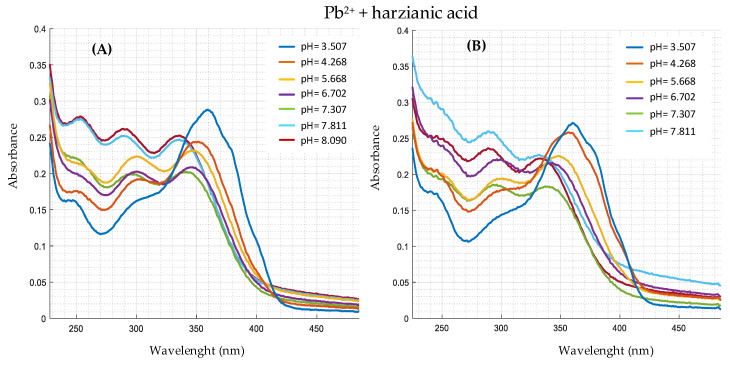
UV-VIS spectra in CH_3_OH/0.1 M NaClO_4_ (50:50 *w/w*) at different pH of: (**A**) $C_{H_{2}L}^{0}$ = 2.0 × 10^−4^ M and $C_{\mathrm{Pb}}^{0}$ = 2.0 × 10^−4^ Pb(ClO_4_)_2_ (ligand to metal ratio 1:1); (**B**) $C_{H_{2}L}^{0}$ = 2.0 × 10^−4^ M and $C_{\mathrm{Pb}}^{0}$ = 4.0 × 10^−4^Pb(ClO_4_)_2_ (ligand to metal ratio 1:2).

The full set of spectrophotometric data collected is shown in Figure 3 (M^2+^ = Cd^2+^), Figure 4 (M^2+^ = Co^2+^), Figure 5 (M^2+^ = Ni^2+^) and Figure 6 (M^2+^ = Pb^2+^), and represents the basis of successive evaluations.

By inspecting the UV-VIS spectra reported above, it is clear that complex species are formed in the M^2+^/harzianic acid solutions. This can be deduced from the fact that UV-VIS spectra not only are substantially different at different pH, but they considerably depend on the ligand to metal ratio and, even more, from the nature of the M^2+^ cation present in the solution [41].

Finally, in order to evaluate the stoichiometry and formation constants of complex species responsible for the observed trend of UV-VIS spectra as a function of pH, and of the ligand to metal ratio, the collected spectrophotometric data, for each metal cation, were processed by the Hyperquad program [42].

This well-known program for the interpretation of equilibrium data fits the experimental data by systematically modifying the equilibrium constants of an assumed set of species to minimize the sum of squared weighted residuals (U).

In Equation (5), A_iK_ is the absorbance measured at the k-th wavelength for the i-th solution, (A_iK_)_c_ represents the absorbance calculated for a fixed set of equilibrium constants, and w_k_ are the weights assigned to each measurement. In the present work, we have assumed $w_{k}\;=\;1$.

(5) $$U\;=\;\Sigma_{i}\Sigma_{k}w_{k}{\left(A_{\mathrm{ik}}-{\left(A_{\mathrm{ik}}\right)}_{c}\right)}^{2}$$

The acid dissociation constants of harzianic acid, which were determined separately and are reported in Figure 1, and the value $K_{w}=\;10^{-14.5}$, for the ionic product of water [43], were separately introduced in the model and kept constant throughout.

Obviously, in whatever solution containing a metal cation and water, side reactions of hydroxo complexes formation may take place and their effect on the experimental data must be considered.

Providentially, the hydrolysis of the M^2+^ cations investigated in this study, in water and in a number of mixed solvents, is well known [44,45,46]. All four hydrated M^2+^ ions of interest here are weak acids, which dissociate producing a number of mononuclear hydroxo complexes, $M{\left(\mathrm{OH}\right)}_{n}^{2-n}$ (n from 1 to 3 for Pb^2+^; n from 1 to 4 for Cd^2+^, Co^2+^, Ni^2+^) and a few polynuclear species, $M_{m}{\left(\mathrm{OH}\right)}_{n}^{2m-n}$ whose stoichiometry may depend on the nature of the M^2+^ cation. This state of affair, when observed on a wide pH range, gives rise to a rather complex equilibrium scenario.

**Figure 7:**
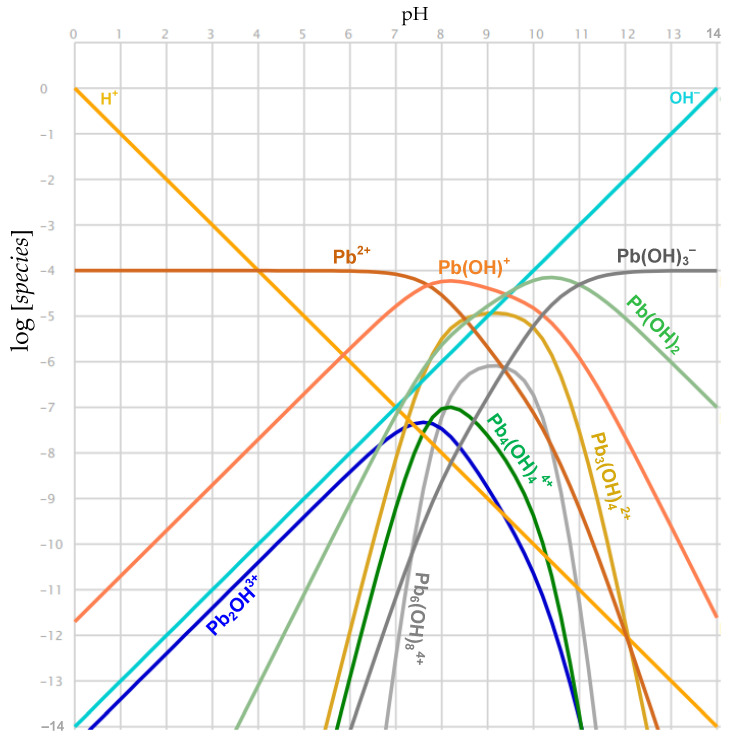
pH scan of a 10^−4^ M aqueous solution of Pb^2+^ based on stoichiometry and formation constants of ${\mathrm{Pb}}_{m}{\left(\mathrm{OH}\right)}_{n}^{2m-n}$ hydroxo complexes gathered from literature [45,46].

For instance, Figure 7 represents what would happen when an aqueous solution, containing 10^−4^ M Pb^2+^, is submitted to a virtual pH scan.

Even a superficial inspection of Figure 7 shows that, at the concentration of metal considered, polynuclear species account only for a small fraction of lead in the solution, so that most of the effect of the side reactions of the hydroxo complexes formation could be accounted for by introducing, in the model, only the conventional $\mathrm{Pb}{\left(\mathrm{OH}\right)}_{n}^{2-n}$ species. However, above pH~8 significant concentrations of ${\mathrm{Pb}}_{3}{\left(\mathrm{OH}\right)}_{4}^{2+}$ are produced. Finally, we hypothesize that, in the mixed (0.1 M NaClO_4_/CH_3_OH, 50/50 *w/w*) solvent used in this study, the situation is not much different from that described in Figure 7. On this basis, we introduced in the model used to fit the spectrophotometric data for lead the hydrolysis species ${\mathrm{PbOH}}^{+}$, $\mathrm{Pb}{\left(\mathrm{OH}\right)}_{2}$, $\mathrm{Pb}{\left(\mathrm{OH}\right)}_{3}^{-}$, and ${\mathrm{Pb}}_{3}{\left(\mathrm{OH}\right)}_{4}^{2+}$, whose formation constants were refined by Hyperquad during the process of minimization of the sum of squared residuals (5) (starting from their values at zero ionic strength taken from reference [46]).

From the point of view of the formation of hydroxo complexes, Pb^2+^ represents the most severe case, since Cd^2+^, Co^2+^, Ni^2+^ are weaker acids, and only the mononuclear $M{\left(\mathrm{OH}\right)}_{n}^{2-n}$ complexes have been considered (since no species $M_{3}{\left(\mathrm{OH}\right)}_{4}^{2+}$ is described and the $M_{2}{\mathrm{OH}}^{+}$ and $M_{4}{\left(\mathrm{OH}\right)}_{4}^{4+}$ polynuclear complexes are clearly irrelevant at the metal concentrations investigated in this study).

Results of Hyperquad processing of spectrophotometric data in Figure 3, Figure 4, Figure 5 and Figure 6 are summarized in Table 2.

Obviously, in order to calculate ${\left(A_{\mathrm{ik}}\right)}_{c}$ in Equation (5), besides refining values of the formation constant for each assumed species, in the process of minimizing the sum of squared residuals, U, Hyperquad also assigns and refines, for each species, a value of the molar extinction coefficient, $\varepsilon_{\lambda }$(M^−1^cm^−1^) at each measured wavelength. By consequence, as a result of data processing by Hyperquad, the $\varepsilon_{\lambda }$ finally assigned to the complex species are obtained.

**Figure 8:**
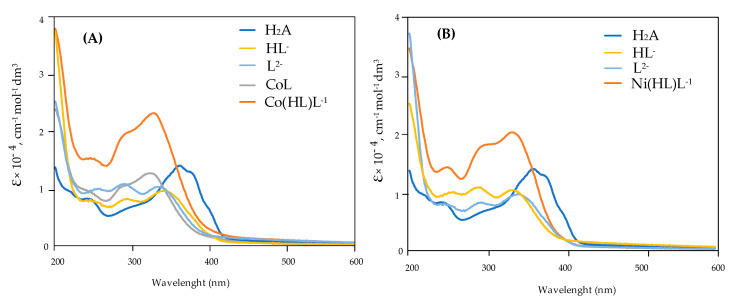
Molar extinction coefficients, $\varepsilon_{\lambda }$ (M^−1^ cm^−1^), of the absorbing species calculated by Hyperquad for the systems: (**A**) Co^2+^-harzianic acid; (**B**) Ni^2+^-harzianic acid.

For instance, the molar extinction coefficients calculated by Hyperquad for all absorbing species in solutions of Co^2+^/harzianic acid and Ni^2+^/harzianic acid are presented in Figure 8.

From Figure 8, it can be seen that the bis-complex M(HL)L^−^ is a very efficient UV absorber, which presumably is responsible for most of the spectral variations in the recorded spectra in Figure 3, Figure 4, Figure 5 and Figure 6.

From data in Table 2, distribution diagrams, showing the fraction of the total metal concentration present in the form of each species, as a function of pH, can easily be drawn for whatever assumed concentration of the metal cation and harzianic acid.

**Figure 9:**
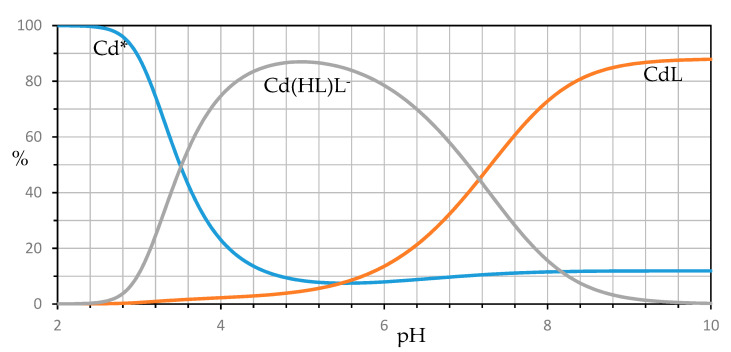
Distribution diagram of species in Cd^2+^-harzianic acid system in CH_3_OH/0.1 M NaClO_4_ (50:50 *w/w*) calculated from formation constants in Table 2 assuming a $C_{\mathrm{Cd}}$ = 1.0 × 10^−3^ M and $C_{H_{2}L}$ = 2.0 × 10^−3^ M (H_2_L = harzianic acid). The symbol Cd* is used to represent all species in the solution that contain the metal cation, but do not contain harzianic acid.

**Figure 10:**
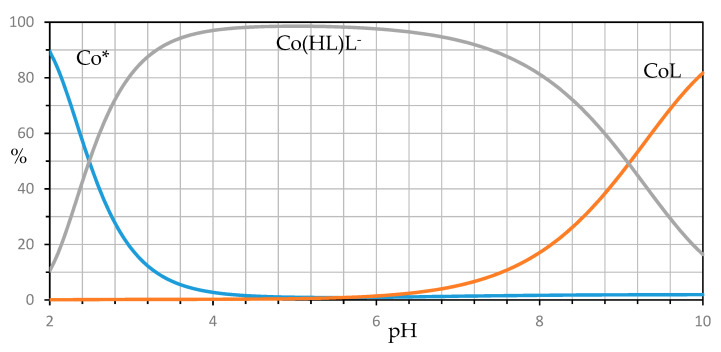
Distribution diagram of species in Co^2+^-harzianic acid system in CH_3_OH/0.1 M NaClO_4_ (50:50 *w/w*) calculated from formation constants in Table 2 assuming $C_{\mathrm{Co}}$ = 1.0 × 10^−3^ M and $C_{H_{2}L}$ = 2.0 × 10^−3^ M (H_2_L = harzianic acid). The symbol Co* is used to represent all species in the solution that contain the metal cation, but do not contain harzianic acid.

For instance, distribution diagrams for M^2+^ = Cd^2+^ and M^2+^ = Co^2+^ are presented in Figure 9 and Figure 10, respectively, from which it can be seen that the bis-complex M(HL)L^−^ is, under the conditions assumed to draw the diagrams, the prevailing species at pH around 6 which is close to the pH of natural ecosystems. However, at the highest pH investigated, M(HL)L^−^ is converted to the 1:1 ML complex, which is the prevailing species at the highest pH investigated. Ni^2+^ represents an exception to this general pattern, since the NiL complex is not formed in the pH range investigated.

Because of differences in stoichiometry of complexes (and, hence, in dimensions of formation constants) and in solvents employed, a direct comparison between the formation constants of M^2+^ complexes with harzianic exposed above and reported apparent affinity constants for other natural chelators is not generally possible or, at best, very uncertain. However, to characterize the efficiency of a ligand, it has been suggested the use of a parameter termed “pM” [47]. Here, we intend pM to represent the minus logarithm (anti-logarithm) of the concentration of the free (solvated) metal cation (i.e., briefly, pM = −log [M^2+^]) in a reference solution of pH = 7.4 containing 1 mM of the ligand and 1 μM total metal concentration. By consequence, a larger pM value corresponds to a lower concentration of the free metal ion in solution at equilibrium and, in principle, to a higher affinity of the relevant ligand for the M^2+^ cation.

For instance, from the above reported formation constants we calculate: pPb = 8.5, pCd = 7.2, pCo = 9.7, pNi = 9.6 when the ligand is harzianic acid. For comparison, pPb = 18.1 is obtained, when the ligand is EDTA in water at zero ionic strength.

Obviously, although in the reference solution, less than 1% of the total metal in solution is found non-bonded to harzianic acid (except for cadmium, for which the metal fraction non-bonded to harzianic acid is about 7%), harzianic acid is a much less efficient chelator than EDTA toward the investigated dipositive metal cations.

**Scheme 2:**
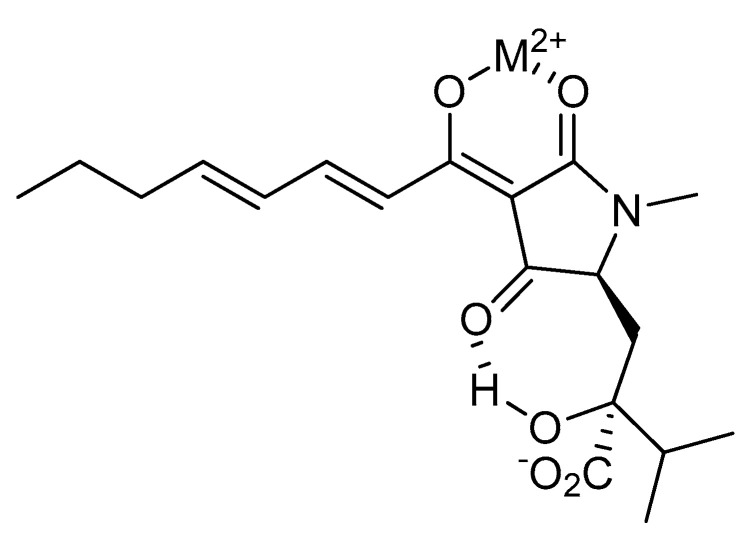
Chelation of metal cations, M^2+^, by harzianic acid.

This is not unexpected, since based on its structure and previous reports, harzianic acid may, at best, be a bidentate chelator toward M^2+^ cations, as are many synthetic products and naturally occurring molecules exposing the 3-acetyl-pyrrolidine-2,4-dione heterocycle. Generally, it is assumed that chelation takes place by the formation of a six membered ring, which includes the metal cation, the amide carbonyl and hydroxyl group on C1. In the case of harzianic acid, this would correspond to the sketch in Scheme 2:

In order to further explore the mode harzianic acid coordinates to M^2+^ cations, ^1^H NMR spectra of harzianic acid in presence of Cd^2+^ and Pb^2+^ were acquired (Figure S5).

In ^1^H NMR spectra particularly interesting are the changes in proton chemical shifts of the hexadienoyl residue and of the pyrrolidine ring in presence and absence of the metal cations.

^1^H NMR spectrum of harzianic acid recorded in presence of CdCl_2_ in CD_3_OD/D_2_O (1:1 *w/w*) showed up-field shifts for H3 (Δδ 0.06) resonating as multiplet at δ 7.52, H4/5 (Δδ 0.05) resonating at δ 7.40 as multiplet, and H5′(Δδ 0.06) resonating as doublet at δ 3.78 (d, J = 9.8 Hz). In the same spectrum, a downfield shift was observed for H2 (Δδ 0.15) resonating as doublet at δ 7.22 (d, J = 16.8 Hz). A similar behavior can be seen in the ^1^H NMR spectrum of harzianic acid recorded in presence of Pb^2+^ ion (Figure S5).

The observed differences on the chemical shifts, obtained by comparison between NMR data of harzianic acid and its metal complexes, confirm the involvement in the coordination complexes of the C = O group of the amide and the external carbonyl groups of the ligand. The 2-carboxy-2-hydroxy-3-methylbutyl group in C5′ seems not to be involved in the coordination because there are no significant differences in the chemical shifts of its protons, and may also be due to the formation of hydrogen bond between the hydroxyl group in C7′ and the carbonyl group in C4′ [48] (Scheme 2). This is in accordance with what was previously reported for tetramic acids, such as tenuazonic acid and analogues [49]. This hypothesis could be also confirmed by the solid-state FT-IR data concerning the complexation between the toxic metals and harzianic acid. In fact, by analysis of the spectra (Figures S6–S10), it is evident a shift of the frequency of the stretching band of the CO and of the C = C towards lower wavenumbers compared to the free ligand, as reported in similar compounds [50].

Our findings show that harzianic acid forms stable chelating complexes with a range of metal ions. Natural ligands containing hydroxyl, carboxylic, and carbonyl groups are structurally suitable to form coordination complexes. In fact, studies conducted on kojic acid [51] and dehydroacetic acid [52] show the formation of complex species with high stability in mixed solvents.

Despite the considerable interest in relation to numerous potential use and applications of chelating compounds, including decontamination of soils, few studies evaluate chelating properties of fungal compounds to find promising candidates for practical applications in biotechnologies. In fact, the contamination of soils with heavy metals represents a challenge because many synthetic chelating agents destroy the soil matrix and remove nutrients. On the contrary, natural compounds might have the capacity to remove heavy metals without deteriorating the soil properties [17]. Furthermore, the possible interest of natural chelating compounds is not exclusively related to the agriculture field, but also in many sections of medicine and biology [21,53].

## 4. Conclusions

In this study, the investigations conducted on the chelating properties of harzianic acid toward some toxic heavy metals show the capacity of this fungal metabolite to form stable neutral or negatively charged coordination complexes in metal/harzianic acid ratio 1:1 or 1:2 depending on the pH conditions. In fact, as can be seen from the distribution diagrams (e.g., Figure 9 and Figure 10), although both species are present, the neutral or charged coordination complexes predominate at different pH ranges.

It seems likely that the biological activities of harzianic acid are related to its complexing ability. In particular, the capacity to form complexes with toxic heavy metals might be important for the decontamination of soils with high heavy metal contents.

## Figures and Tables

**Table 1:** Characterization of M^2+^-harzianic acid complexes by LC-MS. H_2_L represents the full protonated harzianic acid molecule, C_19_H_27_NO_6_.

Ion	Experimental Mass	Formula	Exact Mass
Harzianic Acid + Cd(ClO_4_)_2_			
[H_2_L + H]^+^	366.1915	C_19_H_28_NO_6_	366.1917
[H_2_L + Cd + ClO_4_]^+^	578.0335	C_19_H_27_NO_10_CdCl	578.0331
[2H_2_L − H + Cd]^+^	843.2624	C_38_H_53_N_2_O_12_Cd	843.2632
Harzianic Acid + CoCl_2_			
[H_2_L + H]^+^	366.1913	C_19_H_28_NO_6_	366.1917
[H_2_L + Na]^+^	388.1721	C_19_H_27_NO_6_Na	388.1736
[2H_2_L − H + Co]^+^	788.2910	C_38_H_53_N_2_O_12_Co	788.2931
Harzianic Acid + NiCl_2_			
[H_2_L + H]^+^	366.1910	C_19_H_28_NO_6_	366.1917
[H_2_L + Na]^+^	388.1762	C_19_H_27_NO_6_Na	388.1736
[2H_2_L − H + Ni]^+^	787.2938	C_38_H_53_N_2_O_12_Ni	787.2952
Harzianic acid + Pb(ClO_4_)_2_			
[H_2_L + H]^+^	366.1912	C_19_H_28_NO_6_	366.1917
[H_2_L − H + Pb]^+^	572.1507	C_19_H_26_NO_6_Pb	572.1526
[2H_2_L − H + Pb]^+^	937.3326	C_38_H_53_N_2_O_12_Pb	937.3365

**Table 2:** Summary of M^2+^/harzianic acid (H_2_L) stability constants. **σ** denotes the estimated standard deviation.

M^2+^	Reaction	Log (Formation Constant) ± 3σ
Cd^2+^	Cd^2+^ + L^2^^−^ ⇌ CdL	3.82 ± 0.19
	Cd^2+^ + HL^−^ + L^2^^−^ ⇌ Cd(HL)L^−^	9.13 ± 0.23
Co^2+^	Co^2+^ + L^2^^−^ ⇌ CoL	4.70 ± 0.12
	Co^2+^ + HL^−^ + L^2^^−^⇌ Co(HL)L^−^	11.93 ± 0.06
Ni^2+^	Ni^2+^ + HL^−^ + L^2^^−^ ⇌ Ni(HL)L^−^	11.85 ± 0.12
Pb^2+^	Pb^2+^ + L^2^^−^ ⇌ PbL	4.25 ± 0.5
	Pb^2+^ + HL^−^ + L^2^^−^ ⇌ Pb(HL)L^−^	10.77 ± 0.3

## Data Availability

All the data are reported in the manuscript in the Supporting Materials.

## Supplementary Materials

The following are available online at https://www.mdpi.com/2305-6304/9/2/19/s1, Figure S1: Mass spectrum ESIMS QTOF obtained by LC-MS analysis in ESI positive mode of a solution 1.0 × 10^−3^ M of harzianic acid and 1.0 × 10^−3^ M of Cd(ClO_4_)_2_ in MeOH/H_2_O 50:50 (*w/w*), Figure S2: Mass spectrum ESIMS QTOF obtained by LC-MS analysis in ESI positive mode of a solution 1.0 × 10^−3^ M of harzianic acid and 1.0 × 10^−3^ M of CoCl_2_ in MeOH/H_2_O 50:50 (*w/w*), Figure S3: Mass spectrum ESIMS QTOF obtained by LC-MS analysis in ESI positive mode of a solution 1.0 × 10^−3^ M of harzianic acid and 1.0 × 10^−3^ M of NiCl_2_ in MeOH/H_2_O 50:50 (*w/w*), Figure S4. Mass spectrum ESIMS QTOF obtained by LC-MS analysis in ESI positive mode of a solution 1.0 × 10^−3^ M of harzianic acid and 1.0 × 10^−3^ M of Pb(ClO_4_)_2_ in MeOH/H_2_O 50:50 (*w/w*), Figure S5. ^1^H NMR spectra of harzianic acid (HA) (down), Cd(ClO_4_)_2_: HA (middle) and Pb(ClO_4_)_2_: HA (up) recorded in CD_3_OD/D_2_O (50:50 *w/w*) at 400 MHz. Solvent peaks are removed, Figure S6. Solid-state FT-IR spectrum of harzianic acid, Figure S7. Solid-state FT-IR spectrum of Cd (II)/harzianic acid complex, Figure S8. Solid-state FT-IR spectrum of Co (II)/harzianic acid complex, Figure S9. Solid-state FT-IR spectrum of Ni (II)/harzianic acid complex, Figure S10. Solid-state FT-IR spectrum of Pb (II)/harzianic acid complex.
