# Supplementary material for: Coordination Properties of the Fungal Metabolite Harzianic Acid Toward Toxic Heavy Metals
Source: Toxics. 2021 Jan 20;9(2):19. doi: 10.3390/toxics9020019 (PMC7909447; doi:10.3390/toxics9020019)
Supplement: Supplementary file 1 [file toxics-09-00019-s001.pdf]

toxics

Supplementary Materials:

Coordination Properties of the Fungal Metabolite Harzianic Acid toward Toxic Heavy Metals

Gaetano De Tommaso[1], Maria Michela Salvatore[1], Rosario Nicoletti [2,3], Marina DellaGreca [1], Francesco Vinale [4,5,6], Alessia Staropoli[3,5], Francesco Salvatore[1], Matteo Lorito [3,5], Mauro Iuliano[1*] and Anna Andolfi[1,6*]

1 Department of Chemical Sciences, University of Naples Federico II, Naples 80126, Italy; gaetano.detommaso@unina.it (G.DT.); mariamichela.salvatore@unina.it (M.M.S.); dellagre@unina.it (M.DG); frsalvat@unina.it (F.S.)
2 Council for Agricultural Research and Economics, Research Centre for Olive, Fruit and Citrus Crops, 81100 Caserta, Italy; rosario.nicoletti@crea.gov.it (R.N.)
3 Department of Agricultural Sciences, University of Naples Federico II, Portici 80055 (NA), Italy; matteo.lorito@unina.it (M.L.); alessia.staropoli@unina.it (A.S.)
4 Department of Veterinary Medicine and Animal Productions, University of Naples Federico II, 80137Naples, Italy; frvinale@unina.it (F.V.)
5 Institute for Sustainable Plant Protection, National Research Council, Portici 80055 (NA), Italy
6 BAT Center - Interuniversity Center for Studies on Bioinspired Agro-Environmental Technology, University of Naples Federico II, Portici (NA) 80055, Italy.

Figure S1. Mass spectrum ESIMS QTOF obtained by LC-MS analysis in ESI positive mode of a solution 1.0 x 10^{-3} M of harzianic acid and 1.0 x 10^{-3} M of $Cd(ClO_4)_2$ in MeOH/H_2O 50:50 (*w/w*).

Figure S2. Mass spectrum ESIMS QTOF obtained by LC-MS analysis in ESI positive mode of a solution 1.0 x 10^{-3} M of harzianic acid and 1.0 x 10^{-3} M of $CoCl_2$ in MeOH/H_2O 50:50 (*w/w*).

Figure S3. Mass spectrum ESIMS QTOF obtained by LC-MS analysis in ESI positive mode of a solution 1.0 x 10^{-3} M of harzianic acid and 1.0 x 10^{-3} M of $NiCl_2$ in MeOH/H_2O 50:50 (*w/w*).

Figure S4. Mass spectrum ESIMS QTOF obtained by LC-MS analysis in ESI positive mode of a solution 1.0 x 10^{-3} M of harzianic acid and 1.0 x 10^{-3} M of $Pb(ClO_4)_2$ in MeOH/H_2O 50:50 (*w/w*).

Figure S5. ^{1}H NMR spectra of harzianic acid (HA) (down), $Cd(ClO_4)_2$: HA (middle) and $Pb(ClO_4)_2$: HA (up) recorded in CD_3OD/D_2O (50:50 *w/w*) at 400 MHz. Solvent peaks are removed.

Figure S6. Solid-state FT-IR spectrum of harzianic acid.

Figure S7. Solid-state FT-IR spectrum of Cd (II)/harzianic acid complex.

Figure S8. Solid-state FT-IR spectrum of Co (II)/harzianic acid complex.

Figure S9. Solid-state FT-IR spectrum of Ni (II)/harzianic acid complex.

Figure S10. Solid-state FT-IR spectrum of Pb (II)/harzianic acid complex.

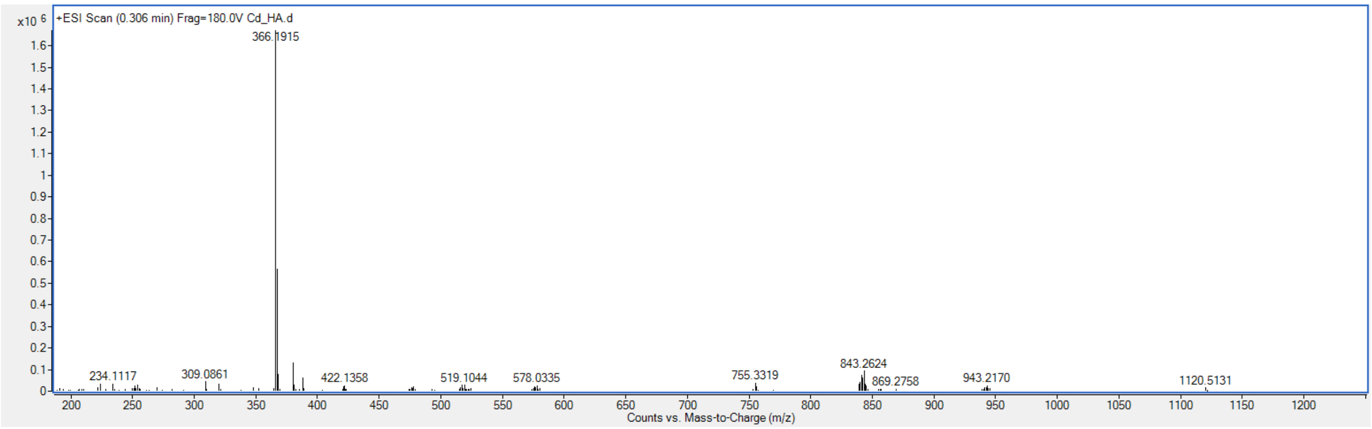

Figure S1. Mass spectrum ESIMS QTOF obtained by LC-MS analysis in ESI positive mode of a solution 1.0 x 10^{-3} M of harzianic acid and 1.0 x 10^{-3} M of $Cd(ClO_4)_2$ in $MeOH/H_2O$ 50:50 (*w*/*w*).

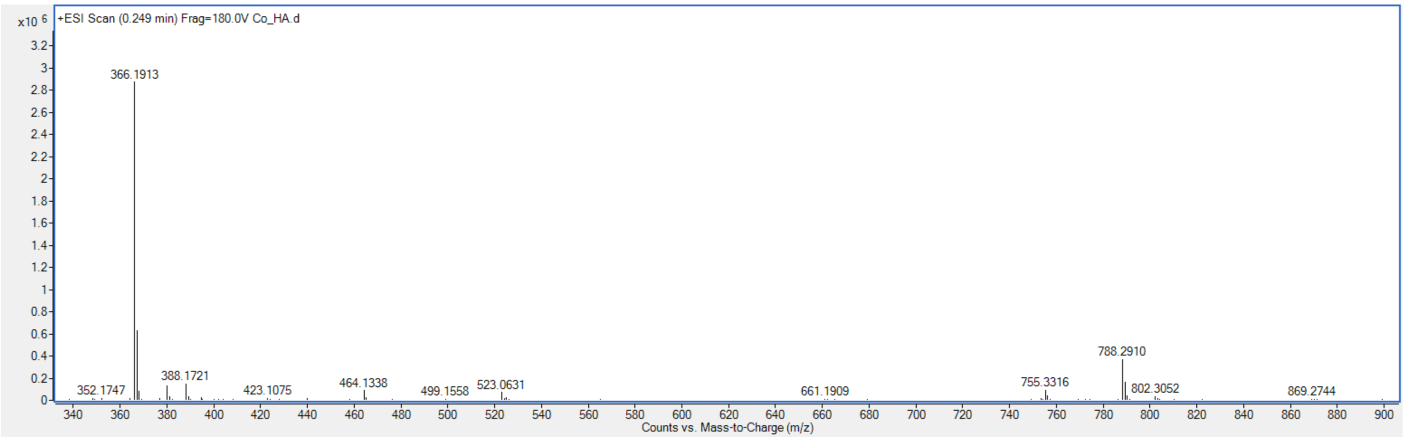

Figure S2. Mass spectrum ESIMS QTOF obtained by LC-MS analysis in ESI positive mode of a solution 1.0 x 10^{-3} M of harzianic acid and 1.0 x 10^{-3} M of $CoCl_2$ in $MeOH/H_2O$ 50:50 (*w*/*w*).

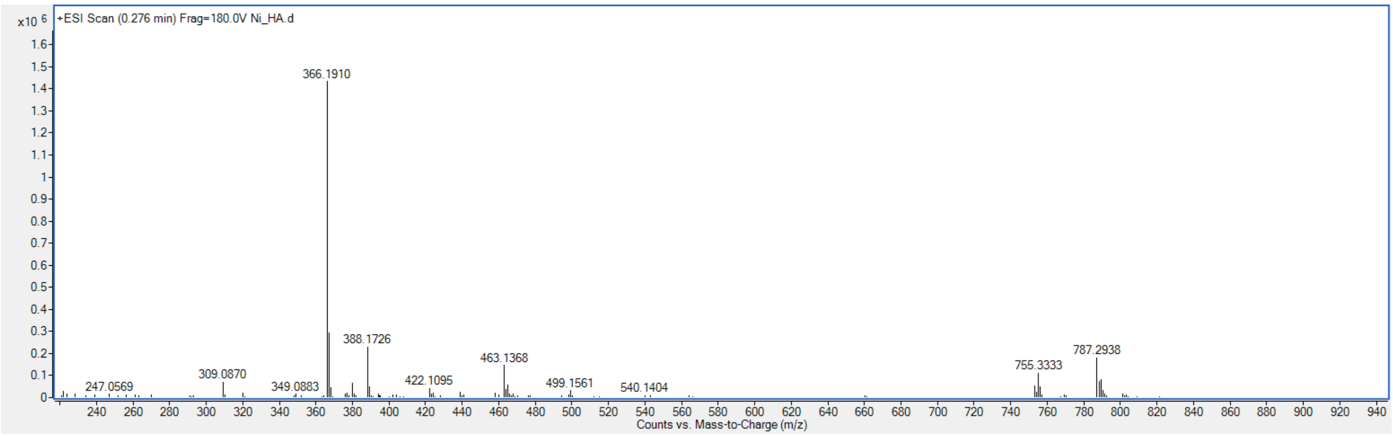

Figure S3. Mass spectrum ESIMS QTOF obtained by LC-MS analysis in ESI positive mode of a solution 1.0 x 10^{-3} M of harzianic acid and 1.0 x 10^{-3} M of $NiCl_2$ in MeOH/H_2O 50:50 (*w*/*w*).

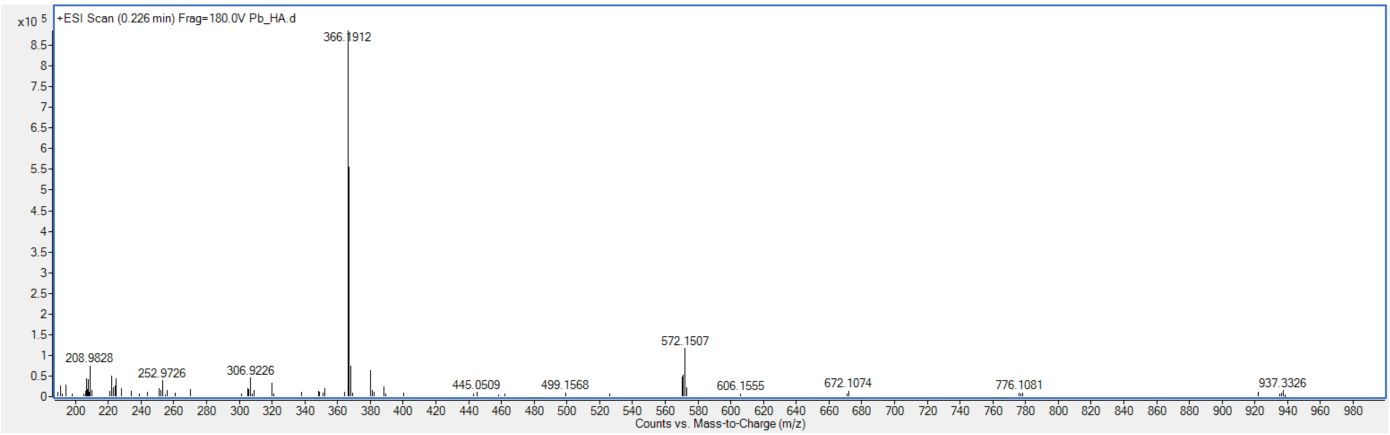

Figure S4. Mass spectrum ESIMS QTOF obtained by LC-MS analysis in ESI positive mode of a solution 1.0 x 10^{-3} M of harzianic acid and 1.0 x 10^{-3} M of $Pb(ClO_4)_2$ in MeOH/H_2O 50:50 (*w*/*w*).

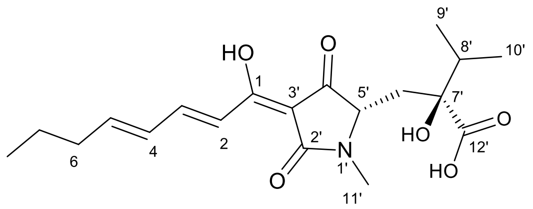

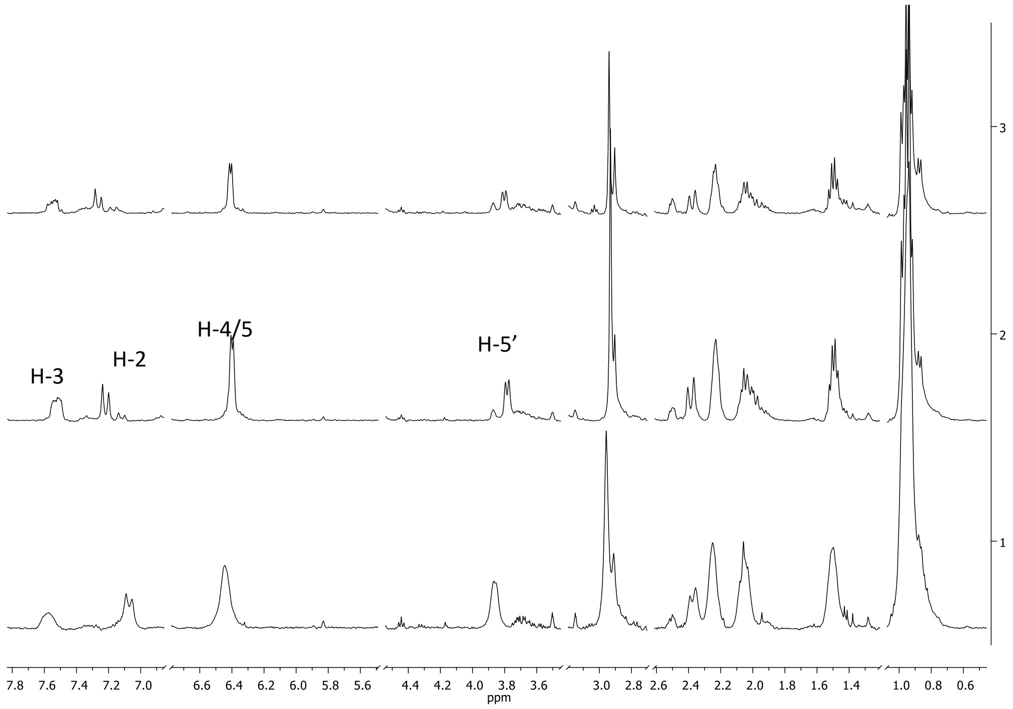

Figure S5. ^{1}H NMR spectra of harzianic acid (HA) (down), $Cd(ClO_4)_2$: HA (middle) and $Pb(ClO_4)_2$: HA (up) recorded in CD_3OD/D_2O (50:50 *w/w*) at 400 MHz. Solvent peaks are removed.

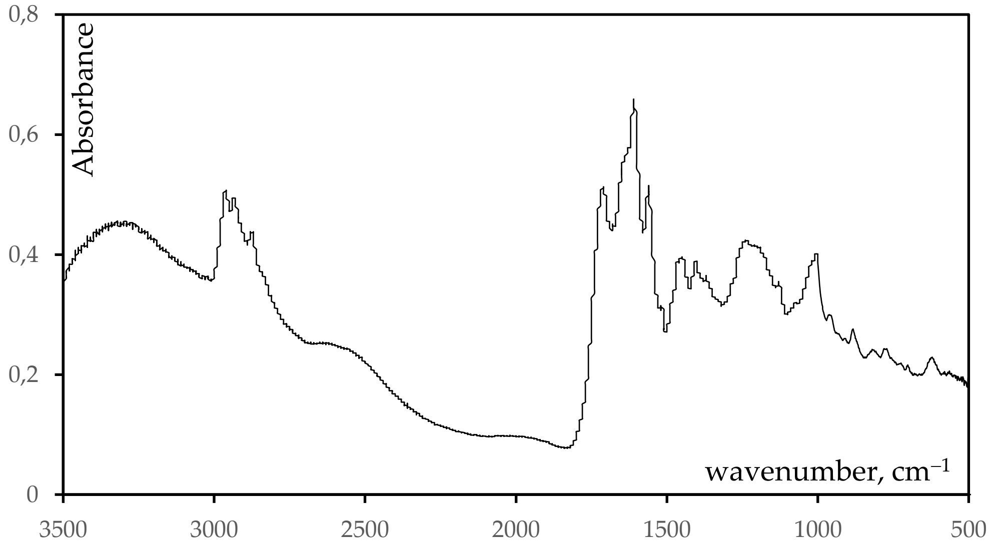

Figure S6. Solid-state FT-IR spectrum of harzianic acid.

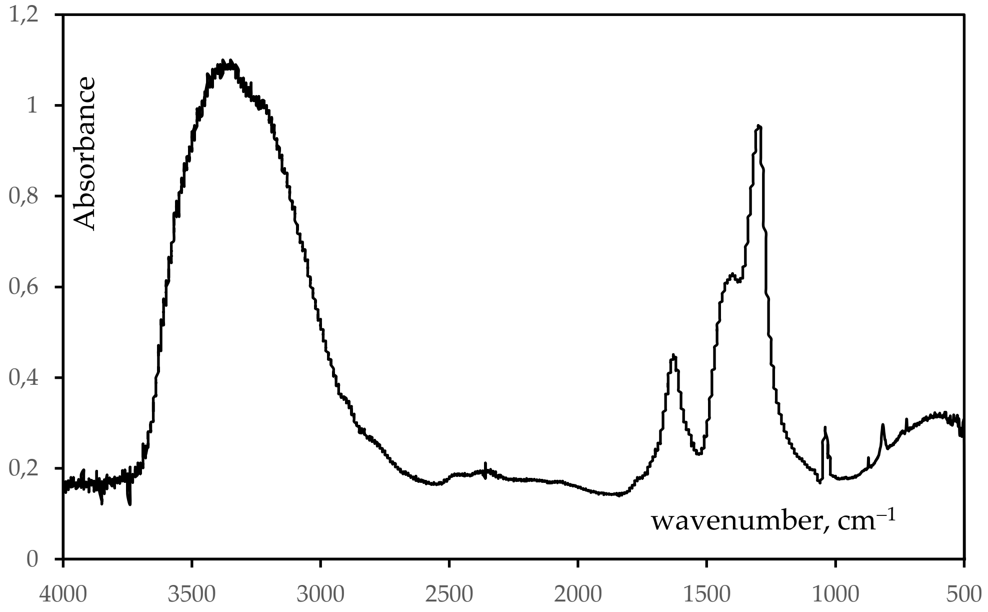

Figure S7. Solid-state FT-IR spectrum of Cd (II)/harzianic acid complex.

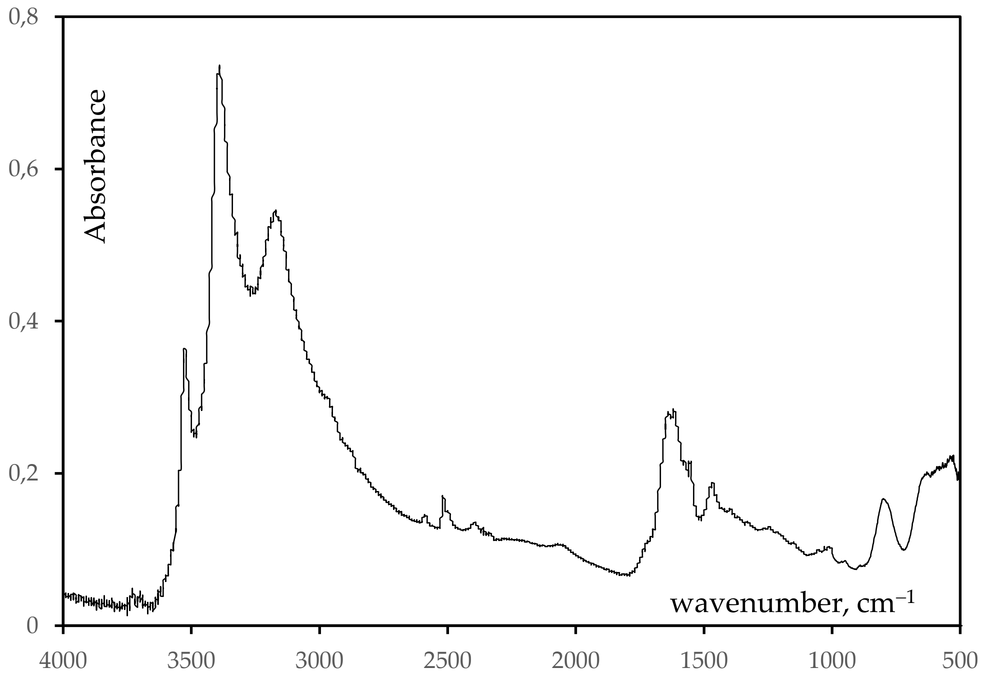

Figure S8. Solid-state FT-IR spectrum of Co (II)/harzianic acid complex.

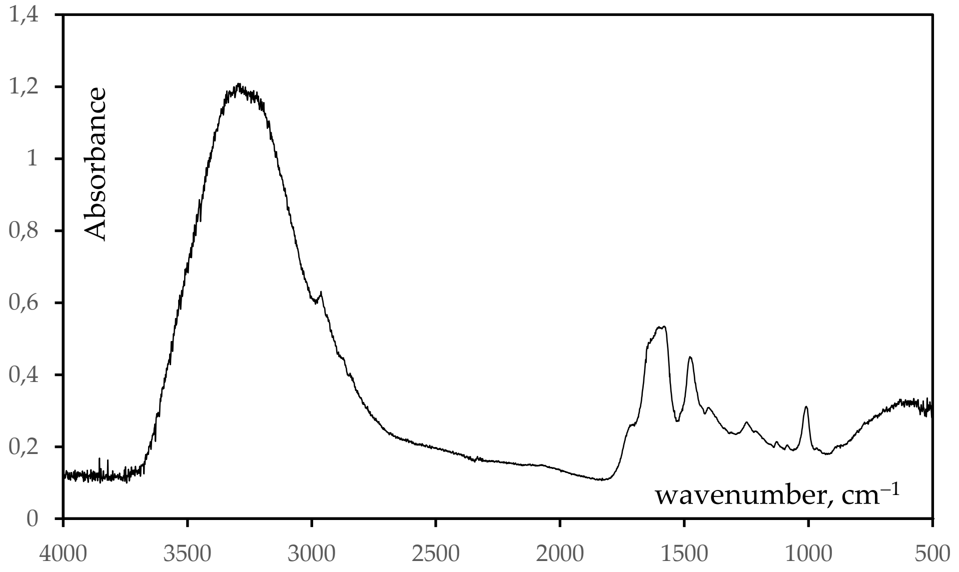

Figure S9. Solid-state FT-IR spectrum of Ni (II)/harzianic acid complex.

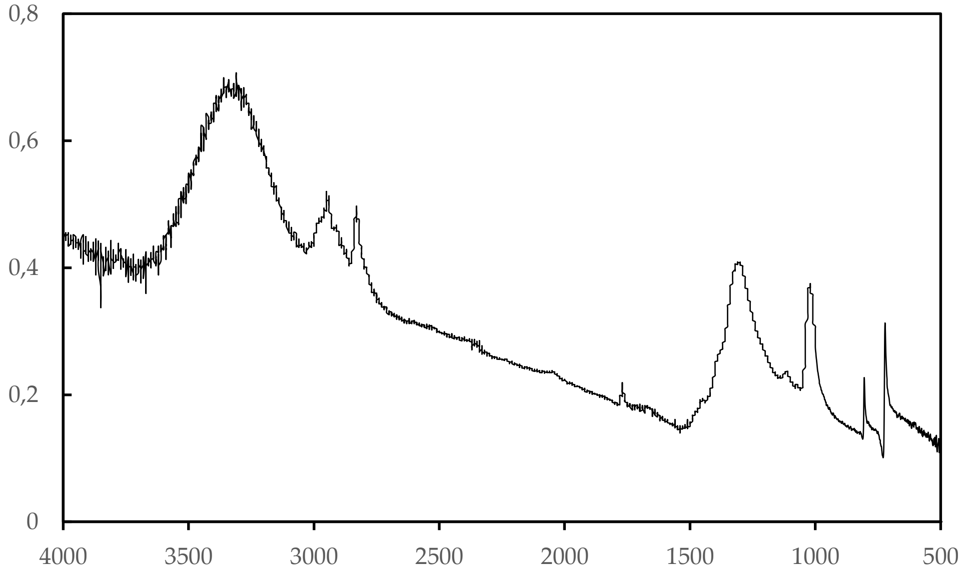

Figure S10. FT-IR spectrum of Pb (II)/harzianic acid complex
